# Modeling Cu(II) Binding to Peptides Using the Extensible Systematic Force Field

**DOI:** 10.1155/2010/724210

**Published:** 2010-03-11

**Authors:** Faina Ryvkin, Frederick T. Greenaway

**Affiliations:** ^1^Department of Chemistry, Emmanuel College, Boston, MA 02115, USA; ^2^Carlson School of Chemistry and Biochemistry, Clark University, Worcester, MA 01610, USA

## Abstract

The utility of the extensible systematic force field (ESFF) was tested for copper(II) binding to a 34-amino-acid Cu(II) peptide, which includes five histidine residues and is the putative copper-binding site of lysyl oxidase. To improve computational efficiency, distance geometry calculations were used to constrain all combinations of three histidine ligands to be within bonding distance of the copper and the best results were utilized as starting structures for the ESFF computations. All likely copper geometries were modeled, but the results showed only a small dependence on the geometrical model in that all resulted in a distorted square pyramidal geometry about the copper, some of the imidazole rings were poorly oriented for ligation to the Cu(II), and the copper-nitrogen bond distances were too long. The results suggest that ESFF should be used with caution for Cu(II) complexes where the copper-ligand bonds have significant covalency and when the ligands are not geometrically constrained to be planar.

## 1. Introduction

Molecular modeling of peptides and proteins interacting with transition-metal ions has the promise of elucidating a wide variety of biophysical phenomena. Such simulations can help establish both the native structure and reaction paths of metalloproteins and explain the roles of metal ions in protein folding and stability [1]. However, the effectiveness of such simulations depends critically on the availability of accurate and computationally reliable force fields for metal ions interacting with proteins. The classical force fields in common use for structure determination using NMR or other internuclear distance data are designed and parameterized for proteins, nucleic acids, and carbohydrates and thus are not optimal for systems with associated metal ions. The extensible systematic force field (ESFF) [2] attempts to provide the widest possible coverage of the periodic table including transition metals with reasonable accuracy and has been successfully used to model several proteins containing metals such as sodium, zinc, iron, and cobalt [2–5]. However only a few studies have been reported for Cu(II) compounds, most of which had severely constrained ligand geometries [3, 4, 6], and ESFF has been used for very few copper-containing proteins or peptides [6–8]. We recently modeled the copper-binding site of lysyl oxidase using a 34-residue peptide homologous to the putative copper-binding region of the enzyme [9] and we now present the results of ESFF computations to model the structure of the copper peptide both to help clarify the structure of the peptide and to investigate the utility of ESFF for Cu(II) compounds where the ligands have few geometrical constraints such as those that are commonly found in bioinorganic complexes.

Lysyl oxidase (EC 1.4.3.13, LOX) is the copper- and quinone-containing amine oxidase that catalyzes the oxidative deamination of peptidyl lysine in elastin and collagen to *α*-aminoadipic-*δ*-semialdehyde, the first step in formation of the cross-links that stabilize these structural proteins [10–13]. Lysyl oxidases from many different sources show a very high level of homology, particularly at the C-terminal end, which contains the copper and quinone binding sites. It has been suggested that the activity of LOX requires one tightly bound Cu(II) atom at its active site [14, 15], although this has been disputed [16]. It is more well established that a Cu(II) is required for posttranslational modification of tyrosine-355 to produce lysyl tyrosyl quinone (LTQ), the lysine-320 linked carbonyl cofactor that is at the center of the active site [17].

LOX is not very soluble except in the presence of high concentrations of urea and is difficult to obtain in large yields. To date the enzyme has not been crystallized, no NMR experiments have been reported, and only limited EPR and CD characterization has been possible [9, 15, 18]; thus almost nothing is known about its tertiary structure. The limited experimental data available suggest that the copper ligands and geometry are similar to those found in the topaquinone-containing copper amine oxidases (CAOs), involving three histidine ligands [19–23]. Krebs and Krawetz [24] utilized various computer prediction models and were first to propose a structural model for the copper-binding site in LOX involving the histidine-rich region between residues 280 and 310 (using the numbering system for the human enzyme). They concluded that three of the four histidines (namely, His 289, 292, 294, 296) are likely copper ligands with the fourth possibly being a general base in the catalytic mechanism. In the present work we have followed up on our spectroscopic study of a Cu(II)-binding LOX-derived peptide containing these residues [9] with ESFF computations to better characterize the metal-binding region of LOX to help identify the copper ligands and to help assess the usefulness of ESFF in metallopeptide and metalloprotein structure predictions.

## 2. Methods and Materials

The amino acid sequence of the 34-residue peptide, P34, chosen for this study includes the putative copper-binding region of LOX [24] and is presented as follows:

  RPRYSWEW${\underline{\text{H}}}^{9}$SA${\underline{\text{H}}}^{12}$Q${\underline{\text{H}}}^{14}$Y${\underline{\text{H}}}^{16}$SMDEFS${\underline{\text{H}}}^{23}$-YDLL DASTQRR-NH_2_.

We previously reported spectroscopic studies of copper binding to P34 [9] where for experimental reasons we replaced residue 11, which in LOX is a cysteine involved in a disulfide bond [25] and unavailable for copper binding, with an alanine. We therefore used the same substitution for this modeling study.

Molecular modeling studies were performed on an *Indigo2 *R10,000 SGI workstation, using a modified [26] *DISGEO* software package with the random metrization Havel algorithm for distance geometry simulation [27], the *InsightII *software package from Biosym. Technologies, Inc. The graphical interface Maestro (Schrödinger Inc, Oregon) was used for further analysis and visualization of generated molecular structures.

Taking advantage of the relatively high speed of the distance geometry procedures, we first used distance geometry calculations to supply starting structures for more sophisticated refinement methods. Initial geometries for the peptide were generated by building up the sequence as an extended conformation using standard amino acid bond lengths, angles, and dihedral angles. These were carried out for the apo-peptide following standard procedures, which have been reviewed [28]. Peptide bonds were constrained to remain in their *trans* configurations. Solvent was not included. The typical input to *DISGEO* included the primary sequence and the distance constraints.

Although we had previously carried out CD and ^1^H NMR experiments for this peptide [9], no secondary structure motifs were experimentally detected and the copper-free peptide adopts a primarily random coil conformation. To simulate the copper-binding site without actually introducing copper, we added distance constraints between histidines selected for modeling as the copper ligands, with the assumption that the copper has three histidine ligands. Since no experimentally based NOE distances were obtained from our NMR studies of the peptide, we instead chose internuclear distances for the simulated copper-binding site based on copper-ligand distances obtained from the literature. For each set of three histidines seven distance constraints, as shown in Table 1, were introduced to force the ligand atoms to be close enough to each other to make copper complex formation possible. Distances between these constrained atoms, which should satisfy triangle inequalities, were then chosen randomly using the random metrization procedure [28]. To obtain geometrically self-consistent data, higher orders of inequalities, such as a tetrangle inequality, might be included, but these are more difficult to implement and are computationally intense for systems involving more than 100 atoms. Thus, the version of the distance geometry program that we used flagged violations only for the triangle inequality.

Crystallographic data for various small inorganic copper complexes with different nitrogen-containing ligands [28–31] were used to establish upper and lower limits on these distance constraints. Copper-histidine nitrogen ligand distances in various copper amine oxidases, thought to have similar copper environments to that in LOX, are typically between 1.9 and 2.1 Å [19–22]. Accordingly, N–N distances for the ligating nitrogens of any two histidines are not more than about 4.2 or 3.0 Å, corresponding to N–Cu–N angles of 180° and 90°, respectively. We chose the upper trans-N–N distance to be 6.0 Å, the maximum permitted by the program and the upper cis N–N distance to be 4.2 Å, to not restrict peptide conformational changes from occurring during the simulation, and the lower N–N distance to be 3.0 Å. This broad range, together with the low number of applied constraints, allowed us a better search for the most favorable conformational geometry. In the copper amine oxidases, histidine has been found to coordinate to copper by both *δ* and *ε* nitrogens [19–22], and since both are possibilities in LOX, this extra distance latitude also allowed either choice to be accommodated. All 18 possible combinations containing any three of the histidine residues present in the peptide sequence (His9, His12, His14, His16, His23) and allowing for all cis and trans isomer combinations were simulated. An example of the numerical constraints is given in Table 1. Analysis of the obtained structures revealed no violations of upper and lower distance restraints. The resulting structures were analyzed for convergence and the conformation with the smallest total error was chosen for further energy minimization calculations.

The coordinates from the best distance geometry structure were used as the initial starting structure for the energy minimization with the copper ion incorporated as a free ion with a charge of +2. The total assembly included 34 amino acids (614 heavy atoms) and one copper ion for the simulation of the protein *in vacuo*. Protonation states of the amino acid side chains were based on standard pKa values and a pH of 7.0. The specific potentials for these geometries were applied for the metal ion according to the ESFF library list (Table 2), but no restrictions were made as to which amino acids could be the copper ligand; so the procedure was expected to explore the whole conformation space. We first carried out energy minimization for the ionic Cu^2+^ option in the ESFF library. Then, taking advantage of the possibilities of the ESFF force field [2] we used that result as a starting point to carry out simulations for the various coordination numbers and geometries of the ESFF library. We tried all ten environments indicated in Table 2 but found that the different geometries for each coordination number resulted in only minor differences in the resulting structures; thus we carried out more extensive analysis only for three cases, namely, four-coordinate copper with C_2v_ symmetry, five-coordinate copper with D_4h_ symmetry, and six-coordinate copper. These represent different coordination numbers (4, 5, and 6) and are of the lowest available symmetry so that restrictions on the metal ion coordination geometry are as low as possible. To investigate the changes to be expected upon reduction, we also carried out the energy minimization for the peptide bound to ionic copper with a +1 charge.

All energy minimization calculations utilized the steepest descent algorithm for the first part of the optimization followed by the conjugated gradient method [32]. The convergence criterion for all runs was set for the maximum derivative to be 0.01 kcal/mol/Å. To check that the structure was not trapped in a false energy minimum, we also adjusted the derivative to 0.001 so that the Cu024 structure was subjected to a much longer energy minimization run. This resulted in a negligible difference in energy, indicating that the lowest possible configuration had indeed been achieved. Nonbonding interactions were evaluated with a cutoff distance of 12 Å and a switch distance of 2 Å. To mimic the aqueous solvent condition, the entire peptide was submerged in a shell of water molecules 10 Å thick, typically containing 1050 water molecules. This water shell alone was first subjected to a minimization and then the optimized solvent system was assembled with the peptide and energy minimization was carried out. No constraints were applied to the peptide-solvent assembly, providing complete freedom of dynamics during simulation for the entire solvated peptide.

## 3. Results and Discussion

**Figure 2:**
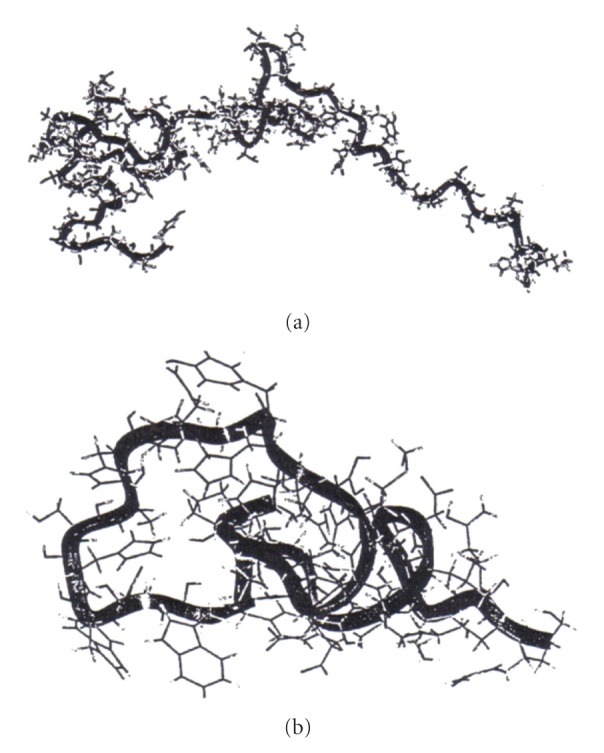
The structures of the apo-peptide before (a) and after (b) distance geometry simulations.

The use of distance geometry simulations resulted in 86 structures showing no violations of upper and lower distance restraints. The conformation with the smallest total error was chosen for further energy minimization calculations. The distance geometry simulation resulted in quick and efficient sampling for a favorable apo-peptide conformation in which the histidine residues thought to be the copper ligands were clustered together (Figure 2). Copper was then added to this site at a position so that the distances from all histidines in question were approximately equal, and the energy minimization procedure was carried out utilizing the ESFF Cu^2+^ parameters. Using this result as a starting point, energy minimization computations were then carried out for ten different copper geometries for Cu(II) chosen from the ESFF library. Although the distance geometry computations had utilized distance constraints to ensure that three histidine ligands were close enough to one another so that all could bind to a copper ion, the energy minimization procedures involved no such constraints and the histidines were free to move away from the copper. Since the different geometries for each coordination number gave very similar structures, we limit our discussion to the representative Cu024, Cu025, Cu026, and the ionic Cu^2+^ cases.

The PROCHECK program [33] was used to assess the overall quality of the minimized geometries. The lowest total potential energies for the simulations for the five types of copper together with an analysis of stereochemical parameters are based on a Ramachandran plot [34]. Given that amino acids bound to copper often have somewhat distorted bond angles, all simulations gave reasonable bond angles and the number of residues in the allowed Ψ-*φ* region of the Ramachandran plot was comparable to or better than typical values calculated using PROCHECK from a crystallographic data base [33]. The structure for the peptide with four-coordinate copper, Cu024, gave the lowest final energy, the fastest convergence, the fewest amino acid residues with unfavorable angles, and the most reasonable bond lengths (Table 3(a)). All minimized structures showed significantly improved stereochemical parameters over the structure produced by distance geometry.

**Figure 3:**
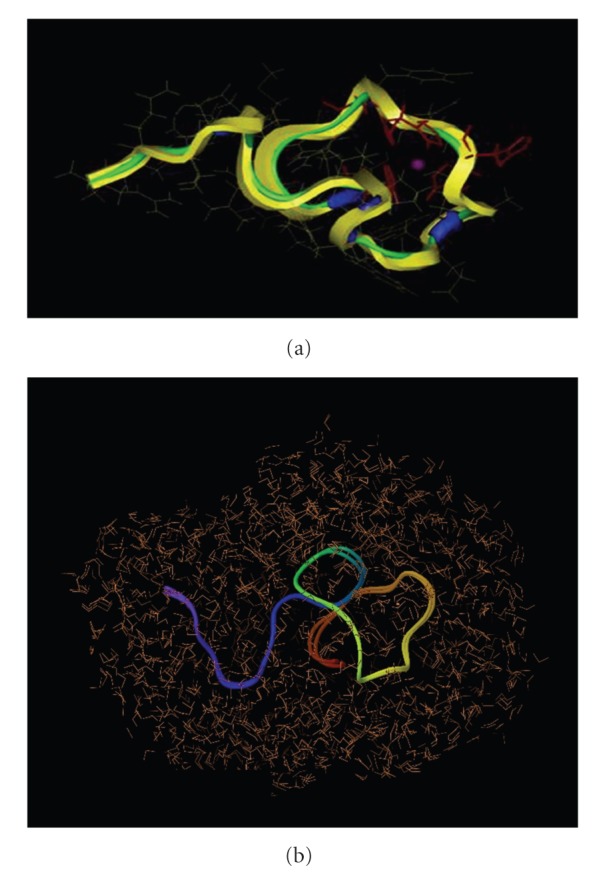
(a) The structure of CuP34 for Cu024 symmetry following energy minimization. The peptide backbone is shown in yellow, random coil regions in green, and *β*-turn regions in blue. Histidine residues are in red. The Cu(II) ion is in magenta. (b) Superposition of CuP34 structures obtained for Cu024 and Cu025 symmetries showing that they have very similar conformations.

Secondary structure analysis of all of the simulated structures revealed the absence of any secondary structure motifs, except for a small amount of *β*-turn (Figure 3(a)), which is consistent with our CD experiments with peptide P34 [9]. The inclusion of the copper ion into the apo-peptide (in any of the geometries we tried) led to minor changes in *β*-turn content but no additional structural motifs. The root mean square deviation (RMSD) value was 0.45 Å for superimposition of the backbone atoms for the solvated and water-free structures confirming that the conformation of the peptide also only underwent minor changes when solvated. In all of our simulations several aromatic residues were exposed to the solvent, consistent with the poor solubility we had observed for the peptide [9].

**Figure 4:**
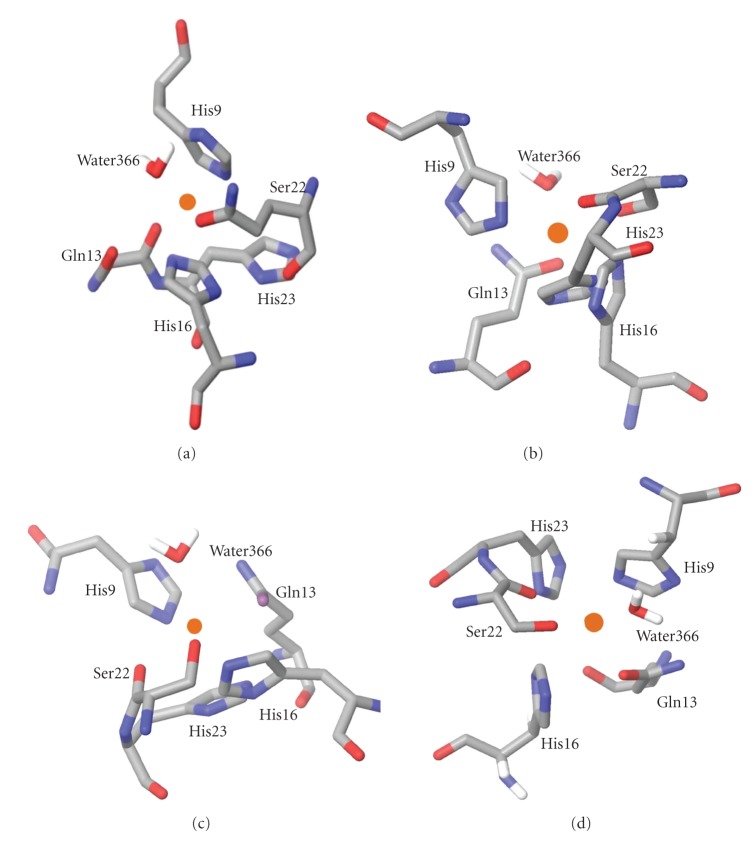
The geometries of the copper sites for the different ESFF coordination numbers: (a) Cu^2+^, (b) Cu024, (c) Cu025, and (d) Cu^1+^.

All simulations resulted in very similar conformations of the peptide backbone (Figure 3(b)) but some variation in the geometry of the copper site. Internuclear distances and bond angles between selected atoms near the copper are summarized in Tables 3(a) and 3(b) and the geometries of the copper sites are shown in Figure 4. The positions of copper and its nearest residues are quite similar for the simulations of Cu024, Cu025, and Cu026 types of the symmetry, but significantly different from the Cu^2+^ simulation.

The results for the Cu^2+^ calculation are shown in Figure 4(a) and Table 3. There are only three ligands within 2.4 Å of the copper, the peptide carbonyl oxygen atom of Ser22 (O1), and the imidazole N*ε* nitrogen atoms of His9 and His16. No other atoms with coordinating ability are within 3.7 Å of the copper although the His23 ring, the side chain amide oxygen of Gln13 (OE1 or O*ε*1), and a solvent-derived water molecule (O366) are in positions that correspond to expected ligand positions for an approximately octahedral geometry around the copper.

**Figure 5:**
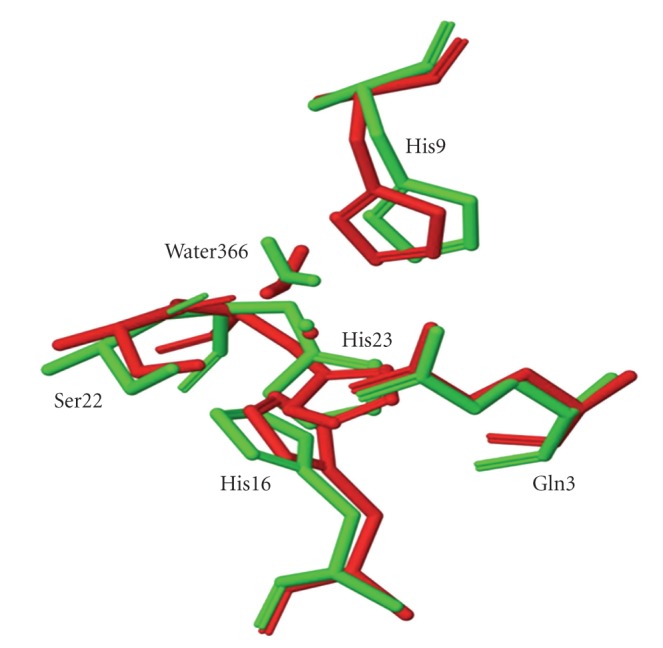
Comparison of the copper sites for the Cu024 (red) and Cu025 (green) structures.

In all other ESFF simulations the immediate coordination environment of the copper ion consists of these same six residues in a distorted octahedral geometry, but with substantially shorter copper-ligand distances for Gln13, His23, and O366. The base of the pyramid corresponds to a quadrangle with unequal sides, with the ligand atoms at the vertices being N*ε* nitrogen atoms from His9 and His16, and oxygen atoms from Ser22 and Glu13. Although the coordinating atoms of three of the basal ligands, N*ε* of His9, O1 of Ser22, and O*ε*1 of Glu13, lie in a plane containing the copper, the fourth, N*ε* of His16, lies slightly above this plane. The two nitrogen and the two carbonyl oxygen atoms are trans to one another (Figure 4) and a water molecule occupies the axial position. In addition, His23 is within bonding distance of the copper in a position opposite to the coordinated water, although it is not oriented correctly for either nitrogen to coordinate to the copper. Thus the geometry is best described as a distorted square pyramid. The Cu025 and Cu026 structures are virtually superimposable and are characterized by copper-ligand bond distances of 2.7–2.9 Å, substantially longer than expected on the basis of crystallographic data for small molecule copper complexes and copper proteins. Although the geometry of the Cu024 site is quite similar to that of the Cu025 and Cu026, sites (Figure 5), the copper-ligand bond lengths for the Cu024 structure are significantly shorter and the Cu–O bond lengths (2.13–2.19 Å) are within the normally expected range of values for Cu–O bonds [35]. The Cu–N bond lengths, however, are still about 0.4 Å greater than normal, which suggests that ESFF computations involving copper may not adequately incorporate the covalent contributions to the bonds with soft nitrogen bases. While the geometry of the Cu024 site is similar to that of the Cu025 and Cu026 sites most of the ligands are at somewhat different orientations.

Because of the lower copper-nitrogen bond lengths, the lower total energy, and the fewer unfavorable amino acid conformations, we consider the Cu024 structure to be the most reliable indicator of the true structure. However, all of the 4-, 5-, and 6-coordinate copper geometries listed in Table 1 gave surprisingly similar results, indicating that the choice of coordination number of the ESFF model is not determinative. Furthermore the coordination geometry chosen for the ESFF field had almost no effect on the final structure. Perhaps most surprising of all was that in every case a five coordinate copper geometry was the end result, with a histidine (His23) in the sixth coordination site, but not oriented correctly to coordinate to the copper. Histidine ligands normally bind fairly symmetrically to the copper since the nitrogen lone pair points towards the copper. The results of our simulations found this to be true for His9, but the histidine ring of His16 was tilted with respect to the copper and that of His23 was almost perpendicular to the correct orientation for bonding to copper. The results indicate that the copper ESFF force fields poorly simulate the orientation dependence of the ligands. Both this result and the long bond lengths found for the Cu–N bonds even in the lowest energy structure indicate that the ESFF deals poorly with the covalency of the copper-ligand bonds. Others have reported that the ESFF force field may inherently overemphasize charge-charge interactions [5] and this seems to be especially important copper.

The inclusion of a solvent-derived water ligand is, however, interesting because, based on comparisons with TPQ-containing CAOs, it is generally believed that LOX has at least one water as a copper ligand. CAOs and LOX are inhibited by cyanide, which displaces the water molecule and binds to the copper site [36, 37]. We previously showed that simulations using the CFF91 force field predict that cyanide can bind instead of water at the axial site [9].

**Figure 6:**
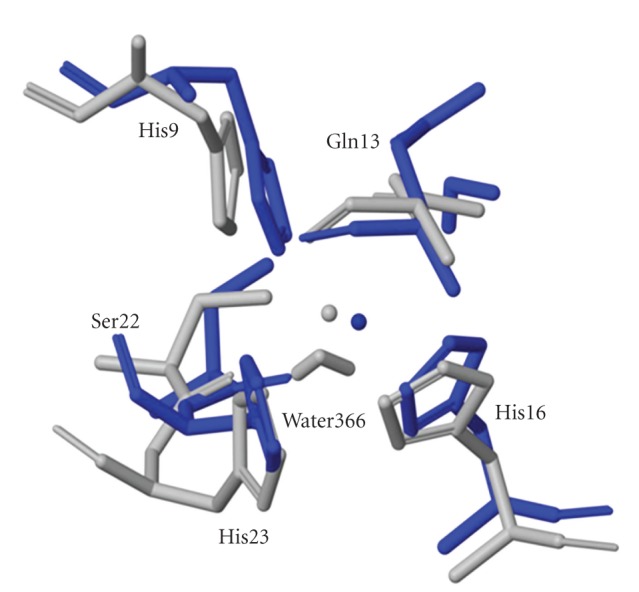
The copper-binding sites for Cu^2+^ (blue) and Cu^1+^ (gray) coordination geometries showing the rearrangement of the histidines upon reduction.

To study the effect of reduction of Cu(II) to Cu(I), we repeated the simulations using the Cu^+1^ field in the ESFF library. This is a significant electrostatic change, resulting in longer copper-ligand bond lengths. The results are summarized in Table 3, and the structure of the copper site for Cu^+1^ is compared with that for Cu^+2^ in Figure 6. As expected, the major changes induced by changing the copper ion charge from +2 to +1 are localized in the vicinity of the copper site (Table 3). His9 and His16 moved away from the copper, while Gln13 and O366 (water) moved about 1.2 Å closer to the copper, and His23 remained outside the bonding sphere (shown in Figure 6). The Cu^+1^ structure most resembled the Cu025 and Cu026 structures.

From our experimental data characterizing the copper site for CuP34 at pH7 [9] the preponderance of evidence suggested the equatorial ligands are three histidine nitrogen atoms and one oxygen atom. Unfortunately no hyperfine structure was observed in the EPR spectra which would have strengthened this assignment of the equatorial ligands. EPR does not give information about the axial ligands. Our ESFF simulations for CuP34 do indicate that three histidine nitrogen atoms (from His9, 16, and 23) are close to the copper, but in all cases one of these (His23) was not oriented correctly to bind to the copper. The imidazole rings of histidines 12 and 14 point out towards the solvent and are not close to the copper in any of the simulations.

The results of this work point to significant problems with the parameterization of the ESFF model for use with Cu(II). The main problem appears to be that the covalent contributions to the copper-ligand bonds are not adequately incorporated with the result that the ligands are insufficiently constrained to ensure that their lone pair orbital points towards the copper, the copper-ligand bond lengths are longer than experimentally found, and there is little difference between the results for the different symmetries for a given coordination number. Alternatively, the parameterization of the angular terms in the potential energy expression for Cu(II) is not well refined. In addition, the Jahn-Teller effect, which invariably leads to longer axial bonds for Cu(II) complexes, is not adequately accounted for. While the ESFF method has been used for a variety of metal complexes, many of these have been for Group IA or IIA metal ions where the bonds are primarily electrostatic and there are no appreciable geometrical constraints on ligands [38]. Very few ESFF studies have been reported for Cu(II) complexes and many of these are for systems with ligands constrained to be planar. Shi et al. [2] studied several porphyrin complexes of the related metal ion, Ni(II), but in these the ligand geometry is also constrained to be planar. Jager and Schilde [3] studied a number of four-coordinate Cu(II) compounds with bidentate ligands coordinating through oxygen atoms and found excellent agreement with crystal structure data. Nicolau and Yoshikawa [4] used ESFF with Cu(II) 8-hydroxyquinolinate complexes, which are bidentate planar aromatic ligands, and Zvimba and Jackson [39, 40] modeled constrained tetradentate ligands. Curtain et al. [6] used ESFF to model Cu(II) in amyloid *β* peptide and Miura et al. [41] modeled Cu(II) coordination to the octapeptide region of prion protein, but in both cases the copper geometry was constrained to be square planar. To our knowledge ESFF has not been used in Cu(II) systems with monodentate ligands with unconstrained geometries, which is a substantially more difficult test of a force field. Accordingly we caution about the use of the ESFF model for Cu(II) complexes involving peptides or any monodentate ligands when the ligand geometries are such that they do not strongly constrain the geometry about the copper ion.

## Figures and Tables

**Figure 1:**
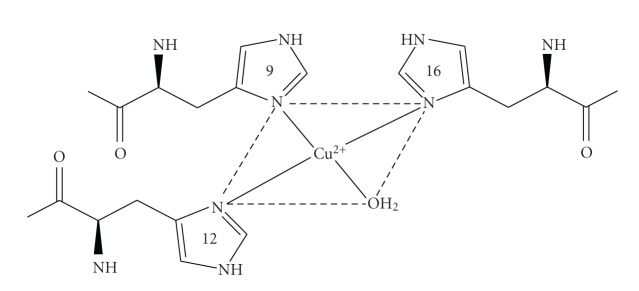
Model of the copper site used to develop the distance constraints that were used for the molecular dynamics calculations for the example shown in Table 1.

**Table 1:** Typical distance constraint input file for distance geometry simulation.

Atom-atom constraints		Lower limit, Å	Upper limit, Å
His9 N*δ*	His12 N*δ*	3.0	4.2
His9 N*δ*	His12 C*ε*	3.0	4.2
His9 C*ε*	His12 N*δ*	3.0	4.2
His9 N*δ*	His16 N*δ*	3.0	4.2
His9 C*ε*	His16 N*δ*	3.0	4.2
His9 N*δ*	His16 C*ε*	3.0	4.2
His12 N*δ*	His16 N*δ*	4.2	6.0

The data are for the structure shown in Figure 1.

**Table 2:** ESFF Library list (*INSIGHT II*) for copper.

1	Cu^+^	Copper^1+^ free ion
2	Cu^2+^	Copper^2+^ free ion
3	Cu024l	Four coordinate copper, C_2v_ symmetry
4	Cu024s	Four coordinate copper, D_4h_ symmetry
5	Cu024t	Four coordinate copper, T_d_ symmetry
6	Cu025	Five coordinate copper
7	Cu025s	Five coordinate copper, C_4v_ symmetry
8	Cu025t	Five coordinate copper, D_3h_ symmetry
9	Cu026	Six coordinate copper
10	Cu026o	Six coordinate copper, O_h_ symmetry

**Table:** (a) Ligand-copper distances (in Å) for different geometries of the copper ion.

Distances for:	Cu^1+^ ion	Cu^2+^ ion	Cu024	Cu025	Cu026
Cu–His16 (N*ε*2)	3.51	2.36	2.39	2.91	2.90
Cu–Wtr366 (O1)	2.44	3.75	2.13	2.7	2.63
Cu–Gln13 (O*ε*1)	2.54	3.74	2.19	2.71	2.68
Cu–His9 (N*ε*2)	2.85	2.34	2.41	2.94	3.08
Cu–His23 (N*δ*1)	3.86	4.69	2.45	3.05	2.94
Cu–Ser22 (O)	2.49	2.17	2.20	2.69	2.72
His16 (N*ε*2)–Wtr366 (O)	4.89	3.93	3.16	5.6	4.88
His9 (N*ε*2)–His16 (N*ε*2)	6.11	4.46	4.67	5.73	5.85
Gln13 (O*ε*1)–Ser22 (O1)	4.83	5.09	4.27	5.20	5.32
His9 (N*δ*1)–Gln 13 (O*ε*1)	4.44	4.89	3.97	4.53	4.44
His23 (N*δ*1)–Ser22 (O1)	3.07	7.04	3.13	2.96	3.14
Ser22 (O1)–Wtr366 (O)	4.36	4.45	3.06	4.27	4.18
His23 (N*δ*1)–Wtr366 (O1)	6.08	7.86	4.44	5.58	5.54
His23 (N*δ*1)–Gln13 (O*ε*1)	5.69	6.37	3.10	4.41	4.34
His16 (N*ε*2)–Ser22 (O)	4.16	2.94	3.07	3.36	3.8
His9 (N*δ*1)–Wtr366 (O)	3.98	5.32	3.14	4.15	3.61
His9 (N*δ*1)–Ser22 (O)	3.93	3.70	3.19	4.45	4.39
His9 (N*ε*2)–His23 (N*δ*1)	3.26	4.11	4.29	4.81	4.83
His23 (N*δ*1)–His16 (N*ε*2)	5.72	4.92	2.30	2.53	2.62
Gln13 (O*ε*1)–Wtr366 (O)	3.09	7.26	3.01	3.50	3.68
His16 (N*ε*2)–Gln13 (O1)	3.17	5.13	2.73	3.33	3.41

**Table:** (b) Ligand-copper angles for different geometries of the copper ion^1^.

Angles (in°) for:	Cu^1+^ ion	Cu^2+^ ion	Cu024	Cu025	Cu026
N16–Cu–O366	109.29	76.32	84.36	108.41	124.13
N16–Cu–N9	147.48	143.12	154.01	156.76	156.46
N9–Cu–O13	65.25	111.3	118.96	106.37	100.33
O13–Cu–O366	76.52	41.17	88.33	80.71	87.64
N23–Cu–N9	55.67	63.04	95.74	107.51	106.28
N23–Cu–N16	101.63	83.38	71.96	50.44	53.45
N23–Cu–O13	51.21	99.28	54.11	100.31	66.69
N23–Cu–O366	148.79	140.31	148.08	156.41	169.19
O22–Cu–O366	124.45	122.43	90.12	105.02	102.62
O22–Cu–N16	85.89	80.87	84.36	90.97	85.04
O22–Cu–O13	97.62	136.03	153.41	148.27	160.31
O22–Cu–N9	94.59	111.10	87.45	104.26	98.24
N23–Cu–O22	52.65	86.66	131.33	62.16	67.21
N9–Cu–O366	97.21	119.77	87.35	94.59	78.11
N16–Cu–O13	82.45	74.71	74.16	73.57	75.31

^1^Refer to Table 3(a) for the exact ligand atoms for column 1.
